# Evolving Surgical Approaches to Adult Perforated Appendicitis: A Systematic Narrative Review

**DOI:** 10.7759/cureus.92225

**Published:** 2025-09-13

**Authors:** Parin Y Patel, Milind K Akhani, Bhavin Baria, Ronak Rathod, Ritesh Patel

**Affiliations:** 1 General Surgery, Health 1 Super Speciality Hospital, Ahmedabad, IND; 2 Surgical Gastroenterology, Hepatopancreatobiliary Surgery and Liver Transplantation, Health 1 Super Speciality Hospital, Ahmedabad, IND; 3 Surgical Gastroenterology, Hepatopancreatobiliary Surgery and Liver Transplantation, Health 1 Hospitals, Jaipur, IND; 4 General Surgery, Shalby Multi-Speciality Hospitals, Ahmedabad, IND; 5 Critical Care Medicine, Health 1 Super Speciality Hospital, Ahmedabad, IND

**Keywords:** appendectomy, intra abdominal abscess, laparoscopic surgery, open surgery, perforated appendicitis, postoperative complications, surgical drains

## Abstract

Perforated appendicitis is the most severe form of acute appendicitis and is associated with significant postoperative morbidity. Advances in laparoscopic surgery and perioperative care have transformed its management, yet the optimal surgical strategy remains debated.

This systematic narrative review, conducted in accordance with Preferred Reporting Items for Systematic Reviews and Meta-Analyses (PRISMA) 2020 and registered in PROSPERO (CRD420251125936), evaluated original studies on adult perforated appendicitis published between January 1, 2000 and June 1, 2025. Six studies encompassing 139,269 patients were included. Three compared laparoscopic and open appendectomy, while others examined prophylactic drainage, timing of drain removal, and immediate versus delayed surgery.

Across studies, laparoscopic appendectomy was associated with shorter hospital stays (4-9.2 vs. 6-10.5 days) and lower overall complication rates (8.3-18.8% vs. 12.5-26.8%) compared with open surgery, though operative times were longer (114-121 vs. 94-106 minutes). Intra-abdominal abscess rates were variable: one early cohort reported similar rates (27.8% vs. 29.2%), the randomized trial showed higher risk with laparoscopy (11.7% vs. 4.5%), and a large database analysis showed lower risk (1.65% vs. 3.57%). Prophylactic drainage did not reduce abscess formation and was associated with increased complications and longer stay, whereas early drain removal following laparoscopy reduced morbidity (3.4% vs. 17.9%) without increasing abscess risk. Immediate surgery, although associated with lower drain utilization (14% vs. 42%), achieved fewer organ-space infections (14.0% vs. 23.8%) and shorter hospital stay (3.1 vs. 9.4 days) compared with delayed surgery.

Overall, the evidence supports laparoscopic appendectomy as the preferred surgical approach for perforated appendicitis, with routine drainage discouraged and early removal favored when drains are placed. Future multicenter randomized studies are needed to refine perioperative strategies and establish standardized best practices in this high-risk subgroup.

## Introduction and background

Perforated appendicitis in adults is a severe surgical emergency that results from full-thickness necrosis and rupture of the appendix, allowing spillage of contaminated contents into the abdominal cavity. This often leads to diffuse peritonitis, intra-abdominal abscess formation, sepsis, and prolonged hospital stay, making it one of the most challenging acute general surgical conditions to manage [1].

Traditionally, open appendectomy was the preferred operative approach for perforated appendicitis, with frequent use of intra-operative measures such as copious peritoneal lavage and prophylactic drains. However, over the past decade, surgical management has evolved with the widespread adoption of minimally invasive techniques and enhanced recovery protocols, both aiming to reduce complications and accelerate recovery [2].

Evidence from randomized and observational studies focused on perforated appendicitis in adults suggests that laparoscopic appendectomy offers comparable or improved postoperative recovery compared to open surgery, with similar rates of intra-abdominal abscess [3]. Recent multicenter data further indicate that routine prophylactic drains do not reduce abscess rates and may increase postoperative morbidity and length of stay [4]. The timing of surgery has also been refined, with early operative intervention shown to shorten hospital stay without increasing postoperative complications [5].

Despite these advances, many studies group perforated appendicitis with other forms of complicated appendicitis, making it difficult to extract perforation-specific trends. This review therefore focuses exclusively on adult patients with perforated appendicitis, drawing on original studies from 2000 to 2025 to clarify current trends in surgical management, including operative approach, timing, and the use of intra-operative adjuncts.

## Review

Method

This systematic narrative review was conducted in accordance with the Preferred Reporting Items for Systematic Reviews and Meta-Analyses (PRISMA) 2020 guidelines [6], aligned with the Synthesis Without Meta-analysis (SWiM) framework [7] and registered in PROSPERO (CRD420251125936).

Eligibility Criteria

We included original studies enrolling adults (≥18 years) with perforated appendicitis, defined as reported by study authors via intra-operative confirmation, histopathology, or radiologic evidence. For studies with mixed severities (e.g., “complicated appendicitis”), inclusion required that outcomes for perforated cases were presented separately and could be extracted without inference. Eligible designs were randomized trials, prospective or retrospective cohort studies, and case-control studies. We required reporting of at least one postoperative outcome of interest.

We excluded pediatric studies; mixed adult-pediatric cohorts without separable adult data; case reports/series with <20 perforated cases; reviews, meta-analyses, guidelines, editorials; conference abstracts without extractable data; non-English publications; and studies published before 2000.

Information Sources and Search Strategy

We searched PubMed, Embase, and the Cochrane from January 1, 2000 to June 1, 2025 (final search: June 1, 2025) using combinations of keywords and Medical Subject Headings (MeSH) related to perforated appendicitis, appendectomy, laparoscopic, open surgery, drain, lavage, irrigation, and timing of surgery. Filters were applied to restrict results to human studies, English language, and the specified time frame. 

Data Items and Operational Definitions

Using a piloted extraction form, two reviewers independently recorded study characteristics, including design, country, setting, study period, and sample size. The following operative strategies were extracted: surgical approach (laparoscopic vs. open; conversions reported where available), timing of surgery (immediate vs. delayed), and drain management (prophylactic drain vs. no drain; early vs. routine removal).

Perforated appendicitis was defined as reported by the original study authors, based on intra-operative confirmation of a full-thickness defect with peritonitis, histopathologic evidence of wall necrosis and rupture, or radiologic signs consistent with perforation. For database analyses, perforation status was accepted according to the coding definitions used in the primary article.

The prespecified postoperative outcomes were assessed as follows.

Intra-abdominal abscess (IAA) was defined according to each study, typically within the index admission or within 30 days postoperatively. Where no time frame was specified, the study definition was accepted without reinterpretation.

Overall postoperative complications were extracted exactly as defined by study authors. When studies explicitly used the Clavien-Dindo classification system (≥ grade II, representing complications requiring pharmacologic, surgical, endoscopic, or radiologic intervention), this definition was recorded. In studies without standardized grading, “overall complications” were reported as author-defined categories, including administrative database codes or grouped clinical outcomes. No attempt was made to reclassify or harmonize these outcomes across studies.

Surgical site infection (SSI) was recorded when reported separately, using study-specific definitions. Length of stay (LOS) was recorded as the mean or median number of days, according to how it was reported in each study. Operative time was recorded as the mean number of minutes, as reported by each study, and readmission was recorded only when it was explicitly reported as occurring within 30 days of the index surgery.

If a study reported results for a broader “complicated appendicitis” cohort, only subgroup data specific to perforated cases were extracted. Where perforation-specific data could not be separated, the study was excluded. Missing or unspecified outcomes were recorded as not reported (NR).

Risk of Bias Assessment

Risk of bias for randomized trials was assessed with the Cochrane RoB 2 tool [8]. Non-randomized studies were appraised with ROBINS-I [9], with confounding domains prespecified to include age, comorbidity burden, severity of contamination (generalized peritonitis vs localized abscess), symptom duration before surgery, and surgeon or center factors. Two reviewers assessed risk independently; discrepancies were resolved by consensus. 

Synthesis Methods

Given heterogeneity in study design, era, patient selection, definitions (particularly for perforation and complications), and outcome capture, no statistical pooling or meta-analysis was performed. In line with SWiM [7], we synthesized results narratively, grouping studies by clinical comparison (laparoscopic vs open, immediate vs delayed surgery, drain vs no drain, early vs routine drain removal) and presenting study-level effect directions and absolute outcome ranges. All numerical results are reported as published by the original studies; no re-estimation, indirect derivation, or imputation was undertaken.

Result

**Figure 1:**
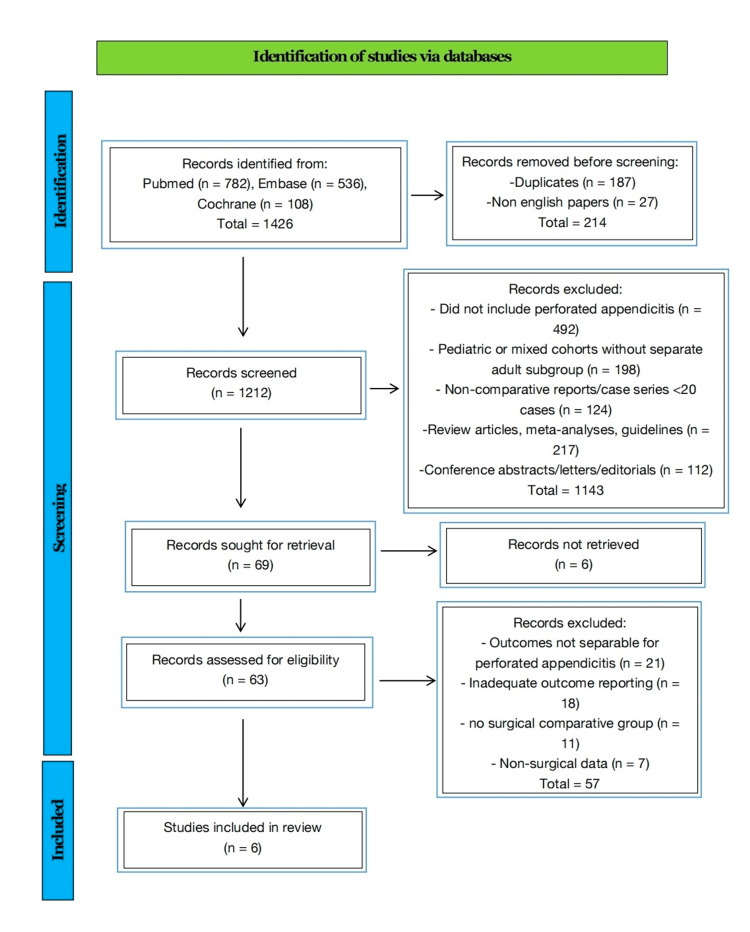
Preferred Reporting Items for Systematic Reviews and Meta-Analyses (PRISMA) 2020 flow diagram for study selection.

The electronic search strategy across PubMed, Embase, and Cochrane identified 1,426 records. After removal of duplicates and non-English publications, 1,212 studies were screened. Following title/abstract screening and full-text review, six studies fulfilled the eligibility criteria and were included in the review. The selection process is summarized in the PRISMA flow diagram (Figure 1).

The included studies comprised three randomized or prospective cohorts and three retrospective database or single-center studies, published between 2001 and 2025, across the USA, Egypt, China, and multicenter international collaborations. The total sample size exceeded 139,000 patients, although this number was disproportionately influenced by a large nationwide database analysis. Key study characteristics are presented in Table 1.

**Table 1:** Characteristics of included studies on surgical management of adult perforated appendicitis. NIS DB = Nationwide Inpatient Sample Database.

Study (Year)	Country	Design	Sample size (total patients)	Study period
Piskun et al. [10] (2001)	USA	Retrospective cohort	52	1993–1999
Masoomi et al. [11] (2011)	USA	Retrospective (NIS DB)	138,184	2006–2008
Talha et al. [3] (2020)	Egypt	Randomized controlled trial	126	2016–2018
Qian et al. [4] (2021)	USA (multicenter)	Post-hoc prospective cohort	634	2015–2018
Fitzgerald et al. [5] (2024)	USA	Retrospective cohort	271	2012–2020
He et al. [12] (2025)	China	Prospective cohort	182	2020–2023

Risk of bias was evaluated using the Cochrane RoB 2 tool for randomized trials and the ROBINS-I tool for observational studies [8,9]. The single randomized controlled trial (Talha et al. [3]) was judged to have some concerns overall, mainly due to issues related to randomization procedures. Among the five observational studies, four were judged to have a moderate overall risk of bias, with confounding identified as the primary limitation. One study (Qian et al. [4]) was judged at serious risk of bias due to residual confounding inherent in its post-hoc design. Detailed assessments are provided in Tables 2, 3.

**Table 2:** Risk of Bias Assessment for Randomized Controlled Trial (RoB 2).

Study	Bias arising from randomization	Bias due to deviations from intended interventions	Bias due to missing outcome data	Bias in measurement of the outcome	Bias in selection of reported result	Overall risk
Talha et al. [3]	Low	Some concerns	Low	Low	Low	Some concerns

**Table 3:** Risk Of Bias In Non-randomised Studies - of Interventions (ROBINS-I).

Study	Confounding	Selection of participants	Classification of interventions	Deviations from intended interventions	Missing data	Measurement of outcomes	Selection of reported result	Overall risk
Piskun et al. [10]	Moderate	Low	Low	Low	Low	Low	Low	Moderate
Masoomi et al. [11]	Moderate	Low	Low	Low	Low	Low	Low	Moderate
Qian et al. [4]	Serious	Low	Low	Low	Low	Low	Low	Serious
Fitzgerald et al. [5]	Moderate	Low	Low	Low	Low	Low	Low	Moderate
He et al. [12]	Moderate	Low	Low	Low	Low	Low	Low	Moderate

Laparoscopic Versus Open Appendectomy

Across the included studies, both laparoscopic appendectomy (LA) and open appendectomy (OA) were reported, with notable variation in practice over time (Table 4). In the earliest report by Piskun et al. [10], LA was attempted in 18 of 52 patients, with 10 conversions to open surgery. In contrast, the large national cohort by Masoomi et al. [11] demonstrated a balanced distribution between LA (n = 69,840) and OA (n = 68,344), reflecting the gradual uptake of laparoscopy in the mid-2000s. The more recent randomized control trial (RCT) from Egypt (Talha et al. [3]) randomized 126 adults to LA or OA, with no conversions reported.

**Table 4:** Surgical approach counts and conversion to open in adult perforated appendicitis. NR = not reported.

Study (year)	Total perforated N	Laparoscopic (n)	Open (n)	Converted to open (n)
Piskun et al. [10] (2001)	52	18	24	10
Masoomi et al. [11] (2011)	138,184	69,840	68,344	NR
Talha et al. [3] (2020)	126	60	66	NR

**Figure 2:**
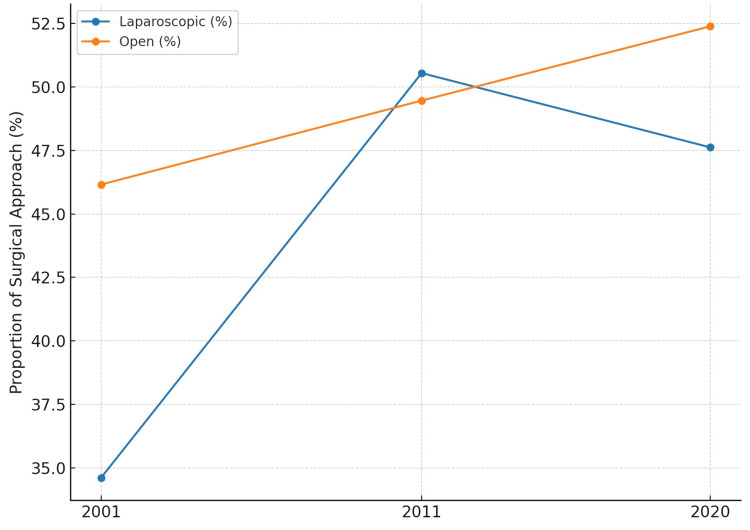
Trends in laparoscopic versus open appendectomy for adult perforated appendicitis (%) across three key studies. Data shown from Piskun et al. [10], Masoomi et al. [11], Talha et al. [3].

In Piskun et al. [10], laparoscopic appendectomy was performed in 34.6% (18/52) and open in 46.2% (24/52) of cases. Masoomi et al. [11] reported a nearly equal distribution, with 50.5% laparoscopic (69,840/138,184) and 49.5% open (68,344/138,184). In Talha et al. [3], laparoscopy accounted for 47.6% (60/126) versus 52.4% open (66/126). These trends are summarized in Figure 2.

Rates of intra-abdominal abscess (IAA) following appendectomy for perforated appendicitis were reported in three studies (Table 5). Piskun et al. [10] found no significant difference between LA (27.8%) and OA (29.2%), while Talha et al. [3] observed a numerically higher but not statistically significant rate with LA (11.7% vs. 4.5%). The large database analysis by Masoomi et al. [11] showed markedly lower absolute IAA rates overall, but consistently higher in OA compared with LA (3.57% vs. 1.65%).

**Table 5:** Intra-abdominal abscess (IAA) after laparoscopic versus open appendectomy in perforated appendicitis. n = number of patients with the outcome; N = total number of patients in that group

Study (year)	Laparoscopic n/N	Open n/N
Piskun et al. [10] (2001)	5/18 (27.8%)	7/24 (29.2%)
Masoomi et al. [11] (2011)	1,152/69,840 (1.65%)	2,440/68,344 (3.57%)
Talha et al. [3] (2020)	7/60 (11.7%)	3/66 (4.5%)

Overall complications and surgical site infection (SSI) were variably reported (Table 6). Piskun et al. [10] described wound infection only, with none after LA compared to 12.5% after OA. In the randomized trial by Telha et al. [3], overall complication rates were lower with LA (8.3%) than OA (22.7%). Similarly, Masoomi et al. [11] reported reduced overall morbidity with LA (18.75%) compared to OA (26.76%).

**Table 6:** Overall complications and surgical-site infections (SSI) in adult perforated appendicitis.

Study (year)	Laparoscopic	Open
Piskun et al. [10] (2001)	0% (SSI)	12.5% (SSI)
Masoomi et al. [11] (2011)	18.75% (overall)	26.76% (overall)
Talha et al. [3] (2020)	8.3% (overall)	22.7% (overall)

Mean length of stay favored laparoscopy, with values ranging from four to 9.2 days compared with six to 10.5 days for OA (Table 7).

**Table 7:** Mean length of hospital stay (LOS) in days after appendectomy for perforated appendicitis.

Study (Year)	Laparoscopic	Open
Piskun et al. [10] (2001)	9.2	10.5
Masoomi et al. [11] (2011)	4	6
Talha et al. [3] (2020)	6.2	8.1

Operative time, however, tended to be longer for LA compared to OA across studies reporting this outcome (Table 8).

**Table 8:** Mean operative time (minutes) in perforated appendicitis. NR = not reported.

Study (Year)	Laparoscopic	Open
Piskun et al. [10] (2001)	114	105.8
Masoomi et al. [11] (2011)	NR	NR
Talha et al. [3] (2020)	120.6	94.0

Timing of Surgery

It was specifically addressed by Fitzgerald et al. [5], who compared immediate versus delayed intervention in 271 adults. Immediate surgery reduced organ-space infections (14.0% vs 23.8%) and shortened hospital stay (3.1 vs 9.4 days) (Table 9).

**Table 9:** Outcomes of Immediate vs Delayed Surgery in Perforated Appendicitis. Data shown from Fitzgerald et al. [5]. NR = not reported, IAA = intra-abdominal abscess

Outcome	Immediate surgery (N=250)	Delayed surgery (N=21)
Drain placement (%)	14.4%	42.9%
Open operation (%)	2.8%	23.8%
IAA (%)	14.0%	23.8 %
Mean operative time (min)	64.1	83.1
Mean LOS (days)	3.1	9.4
Overall complications	NR	NR

Drain Management Strategies

The role of drains was investigated in two studies. Qian et al. [4] analyzed 634 patients and found prophylactic drains offered no reduction in IAA (7% vs 8%), but were associated with higher overall complications (43% vs 28%) and longer hospital stay (four versus three days) (Table 10).

**Table 10:** Outcomes of prophylactic drain use after appendectomy for perforated appendicitis. Data shown from Qian et al. [4]. IAA = intra-abdominal abscess, LOS = length of stay, SSI = surgical site infection

Outcomes	Drain (N=159)	No drain (N=475)
IAA within 30 days	7%	8%
Overall complications	43%	28%
LOS, days	4	3
SSI / secondary interventions	1%	2%

He et al. [12] compared early (≤48h) versus routine (>48h) drain removal in 182 patients, reporting lower IAA (3.4% vs 11.6%), fewer complications (3.4% vs 17.9%), and shorter LOS (four versus six days) in the early removal group (Table 11).

**Table 11:** Outcomes of early versus routine drain removal after laparoscopic appendectomy for perforated appendicitis. Data shown from He et al. [12]. IAA = intra-abdominal abscess, LOS = length of stay, SSI = surgical site infection

Outcomes	Early (≤48 h) removal (N=87)	Routine (>48 h) removal (N=95)
IAA	3.4%	11.6%
Overall complications	3.4%	17.9%
Superficial SSI	0%	2.1%
LOS, days	4	6
Readmission ≤30 days	2	4

**Figure 3:**
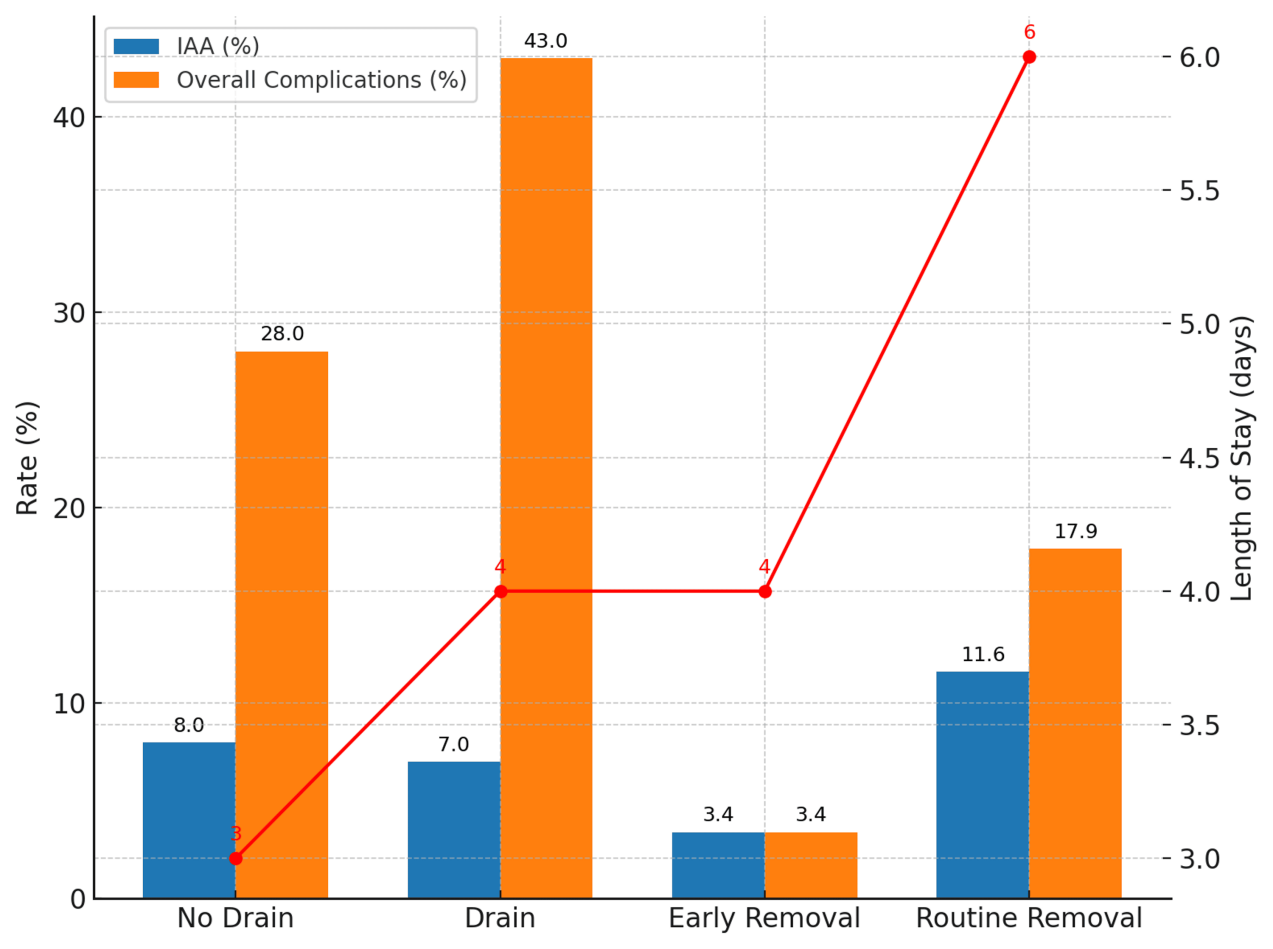
Comparision of outcomes of drain management strategies in perforated appendicitis. Data shown from Qian et al. [4] and He et al. [12]. Length of stay (LOS, days) is represented by the red line. IAA = intra-abdominal abscess

Drain management was assessed in two studies. Qian et al. [4] compared prophylactic drains versus no drains, while He et al. [12] compared early versus routine drain removal. Both studies highlighted that routine or prolonged drainage was associated with higher complication rates and longer hospital stay. These outcomes are visually summarized in Figure 3.

Discussion

The management of perforated appendicitis in adults has changed significantly over the past two decades, and the included studies illustrate how surgical practice has evolved during this time. Early reports reflected considerable uncertainty about the feasibility and safety of laparoscopy in contaminated fields. Piskun et al. [10], in a retrospective cohort from the 1990s, reported that more than half of attempted laparoscopic cases required conversion to open surgery, and intra-abdominal abscess rates were comparable between the two approaches. These findings reinforced the prevailing concern at that time that laparoscopic surgery might expose patients to higher infectious risks in the setting of peritonitis.

By the following decade, larger database studies offered a different perspective. Masoomi et al. [11], using the U.S. Nationwide Inpatient Sample, demonstrated that laparoscopy was being performed in nearly half of perforated appendicitis cases and was associated with shorter length of stay and lower overall complications compared with open surgery. Although intra-abdominal abscess remained a debated outcome, the study indicated that laparoscopy was not only feasible but could provide tangible benefits at the population level. This represented a shift in surgical confidence and paved the way for broader adoption of minimally invasive techniques.

The most definitive evidence emerged with the randomized controlled trial by Talha et al. [3], which confirmed that laparoscopic appendectomy is associated with fewer complications and shorter hospitalization compared with open surgery, despite somewhat longer operative times. While the study found a slightly higher but non-significant incidence of abscess after laparoscopy, this did not offset the overall benefits in morbidity reduction. These results aligned with global surgical trends, where laparoscopy has become the standard approach even in the context of complicated intra-abdominal infection. Together, these studies demonstrate the progression from early technical difficulty and high conversion rates to more modern evidence supporting laparoscopy as the preferred surgical strategy for perforated appendicitis.

The question of surgical timing has also been revisited in recent years. Traditionally, delayed or interval appendectomy was sometimes advocated to allow sepsis to resolve before surgery. However, Fitzgerald et al. [5], in a multicenter cohort, showed that immediate appendectomy was associated with shorter hospital stay and reduced drain use compared with delayed surgery, without increasing postoperative complications. These findings are consistent with the principle of early source control in sepsis management and suggest that delaying surgery offers little benefit in the modern era. Nevertheless, this was a single retrospective study, and multicenter randomized evidence is needed to confirm whether these results are generalizable across different healthcare systems and patient populations.

The role of drains has historically been one of the most controversial aspects of managing perforated appendicitis. For decades, drains were routinely used on the assumption that they would reduce the risk of abscess formation. However, Qian et al. [4], in a post-hoc analysis of a multicenter prospective cohort, demonstrated that prophylactic drains did not reduce intra-abdominal abscess rates and were associated with higher complication rates and longer length of stay. This finding challenges long-standing surgical dogma and aligns with evidence from other intra-abdominal procedures, where routine drains have increasingly been abandoned. Complementary evidence from He et al. [12] adds further nuance: in patients undergoing laparoscopic appendectomy with drains placed, early removal within 48 hours reduced both complications and length of stay compared with routine delayed removal. Taken together, these findings suggest that drains should not be placed prophylactically and, if used, should be removed early rather than left in place routinely.

While the direction of evidence favors laparoscopy, early surgery, and selective drain use, important limitations must be acknowledged. The definition of perforation varied across studies, including intra-operative, radiologic, and histopathologic confirmation, which limits comparability. Outcome reporting was also inconsistent. Two studies (Talha et al. [3] and He et al. [12]) explicitly defined overall complications according to the Clavien-Dindo classification (≥ grade II), whereas others reported broader, author-defined categories. This inconsistency restricts the ability to compare complication rates directly. Furthermore, intra-abdominal abscess was reported with differing time frames, ranging from 30-day readmissions to index hospitalization, which again complicates interpretation.

Another limitation is the disproportionate weight of large administrative databases. The Masoomi et al. [11] study contributed the vast majority of patients in this review. While these data provide valuable population-level insights, they are constrained by the accuracy of coding and the inability to account for clinical factors such as peritoneal contamination severity or surgeon expertise. Smaller prospective and randomized studies provide richer clinical detail but lack statistical power. This imbalance highlights the importance of contextualizing results rather than pooling them indiscriminately, a key reason why narrative synthesis rather than meta-analysis was adopted.

The relative scarcity of perforation-specific studies also deserves attention. Despite the frequency of appendicitis worldwide, only six studies met criteria for inclusion over a 25-year period. Many clinical studies still group perforated appendicitis with other forms of complicated appendicitis, such as phlegmon or localized abscess, which have distinct prognoses and management implications. This lack of granularity continues to limit the evidence base and underscores the need for more targeted research on perforated cases specifically.

From a clinical perspective, the synthesis of available evidence indicates several practical implications. Laparoscopic appendectomy should be considered the operative standard for perforated appendicitis in adults, providing superior short-term outcomes compared with open surgery. Prompt surgery appears to reduce morbidity and resource utilization compared with delayed approaches. Routine prophylactic drainage is not beneficial and may be harmful, while early removal of drains, when used, reduces complications and length of stay. These findings reflect a broader surgical trend toward minimally invasive, early, and streamlined management in intra-abdominal sepsis.

Future research should address several important gaps. Standardized definitions of perforation and outcomes, particularly consistent use of the Clavien-Dindo classification, are needed to improve comparability across studies. Randomized multicenter trials are warranted to validate the optimal timing of surgery and to clarify the role of drain management strategies. Beyond traditional surgical outcomes, future studies should also incorporate quality of life, return to work, and cost-effectiveness to better capture the broader impact of management strategies. Finally, given that most available data derive from the United States and select international centers, expanding research to include low- and middle-income countries would improve global applicability.

## Conclusions

The surgical management of perforated appendicitis in adults has evolved toward minimally invasive, early, and selective strategies. Laparoscopic appendectomy now provides shorter hospitalization and lower complication rates compared with open surgery, without a consistent increase in intra-abdominal abscess. Immediate surgery appears advantageous over delayed intervention, and routine drainage is not supported, with early removal favored when drains are used. Future multicenter randomized studies are needed to refine these strategies and establish standardized best practices for this high-risk condition.
